# Advanced Squamous Cell Carcinoma of Gall Bladder Masquerading as Liver Abscess With Review of Literature Review on Advanced Biliary Tract Cancer

**DOI:** 10.7759/cureus.16867

**Published:** 2021-08-04

**Authors:** Syed Hamza Bin Waqar, Navid Salahi, Li Zhonghua, Isabel M McFarlane

**Affiliations:** 1 Internal Medicine, State University of New York (SUNY) Downstate Medical Center, New York, USA; 2 Pathology, State University of New York (SUNY) Downstate Medical Center, New York, USA; 3 Pathology, Kings County Hospital Center, New York, USA

**Keywords:** advanced biliary tract cancer, gallbladder cancer, carcinoma, squamous cell cancer, abc, gbc

## Abstract

Advanced biliary tract carcinoma (ABC) tends to have a poor prognosis, with trials done having limited data from oncologists' perspectives. Squamous cell variant of gallbladder cancer (GBC) is one of the rarest forms of cancer known in the literature, with a very aggressive course and dismal prospects. Herein, we present a case of a 67-year-old man who got diagnosed with squamous cell carcinoma, which initially masqueraded as liver abscess and was associated with severe hypercalcemia, pyrexia, jaundice, and submassive pulmonary embolism.

## Introduction

Gallbladder carcinoma is afflicted with unfavorable outcomes in comparison to other biliary tract carcinomas. Most cases are adenocarcinoma or adenosquamous variant, with pure squamous cell carcinoma accounting for less than three percent. Squamous carcinoma is associated with an aggressive invasive pattern with limited lymph node involvement, disproportionate to tumor size [1,2]. Given the proximity to the gastrointestinal tract, it can involve the stomach, duodenum, or transverse colon, or hepatic parenchyma [1-3]. Herein, we present a case of a 67-year-old man who presented to the emergency department with progressive fatigue, unintentional weight loss, jaundice, and hypoxia. He got diagnosed with pulmonary embolism and, on further investigation, found to have a massive gallbladder mass invading the liver, mimicking liver abscess. On biopsy, the mass was proven to be pure squamous cell carcinoma. Treatment with chemoradiation was discussed with the patient but later deferred as he opted for hospice care. 

## Case presentation

A 67-year-old man, a known Jehovah's witness, presented to our tertiary care setup with progressive worsening of fatigue, anorexia, dull generalized abdominal pain with frequent bloating and constipation, and unintentional 55 pounds weight loss over two months. Associated with these changes was increasing shortness of breath with the slightest exertion, two months preceding the worsening fatigue. In the emergency department, the patient was febrile with 100.7F, blood pressure being 89/60 mmHg, tachypnea with a respiratory rate of 23 breaths per minute, resting tachycardia to 110 beats per minute, and low oxygen saturation to 90% on room air which worsened to 86% on minimal exertion. On physical exam, the patient appeared tired, had scleral icterus, triceps wasting, dry mucous membranes, and abdominal tenderness to palpation over all quadrants, most pronounced in the epigastrium and right upper quadrant.

**Figure 1:**
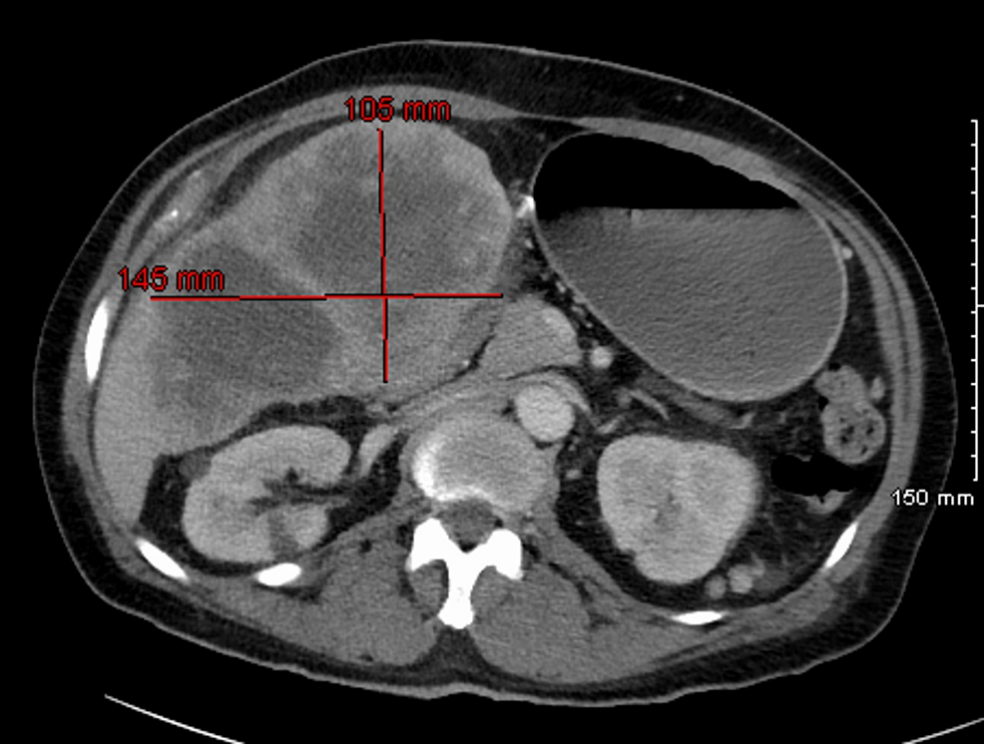
CT Abdomen (axial view) showing a large heterogeneous mass which appears continuous with the gallbladder and liver. The mass likely extends from the gallbladder fundus into the liver parenchyma. The mass is peripherally enhancing with central hypoenhancement having multiple patchy areas of enhancement. The mass measures 105 x 145 x 154 millimeters in AP, transverse, and craniocaudal dimensions. CT: computed tomography.

**Figure 2:**
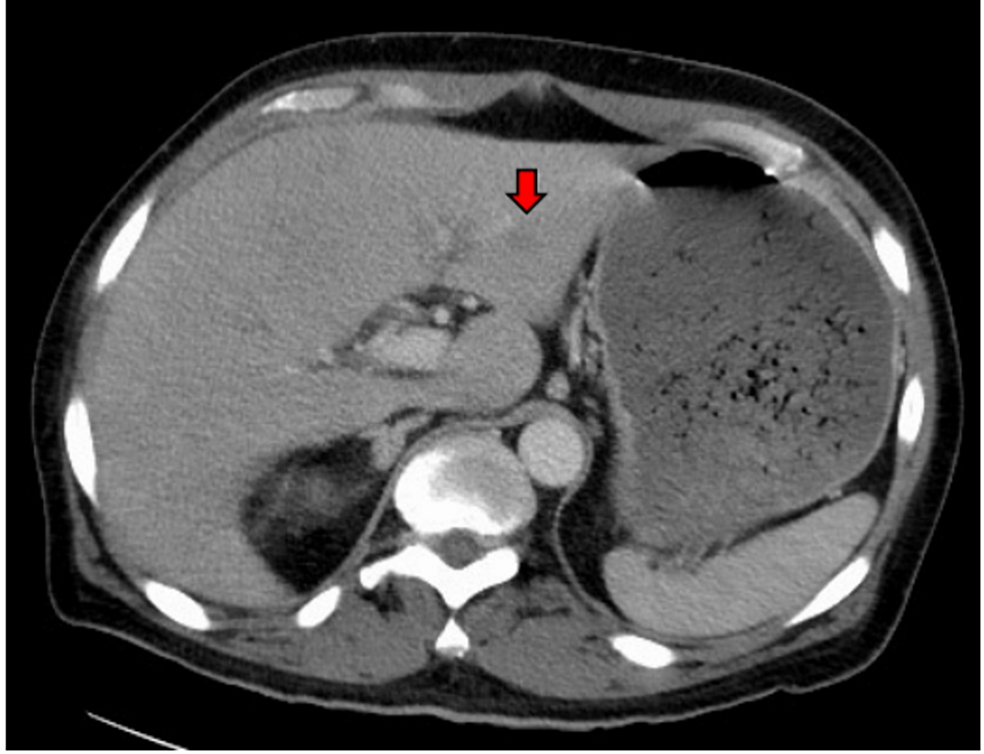
CT abdomen (axial view) showing small ill-defined hypoattenuating area within the left lobe of the liver in segment III measuring 12 x 13 x 15 millimeters (shown with a red arrow). CT: computed tomography.

Complete blood count and comprehensive panel were obtained on initial labs, which were significant for hemoglobin of seven grams per deciliter (g/dl) and borderline normocytic anemia with poikilocytosis and occasional target cells, normal leukocyte and platelet count; elevated blood urea nitrogen to creatinine ratio and severe hypercalcemia to 14.2 milligrams per deciliter. Given hypoxia at room air, the patient was sent for computed tomography (CT) of the chest showed submassive pulmonary embolism with extension to segmental and subsegmental vessels bilaterally. In the emergency department, the patient was given boluses of normal saline with calcitonin, zoledronic acid, therapeutic low molecular weight heparin, and oxygen delivery optimized with the nasal cannula. The patient was admitted to the medicine floor for further optimization. Given the concern of malignancy or chronic inflammatory process, CT abdomen/pelvis obtained showed large heterogenous mass extending from gallbladder's fundus and invading liver parenchyma with peripheral enhancement and central hypo-enhancement with contrast measuring 105 x 145 x 154 mm in anteroposterior and craniocaudal dimensions suggestive of a tumoral mass with extensive necrosis or liver abscess. There were also perihepatic ascites, and a satellite lesion in the left hepatic lobe measuring 12 x 13 x 15 mm noted on imaging (Figures 1, 2).

**Figure 3:**
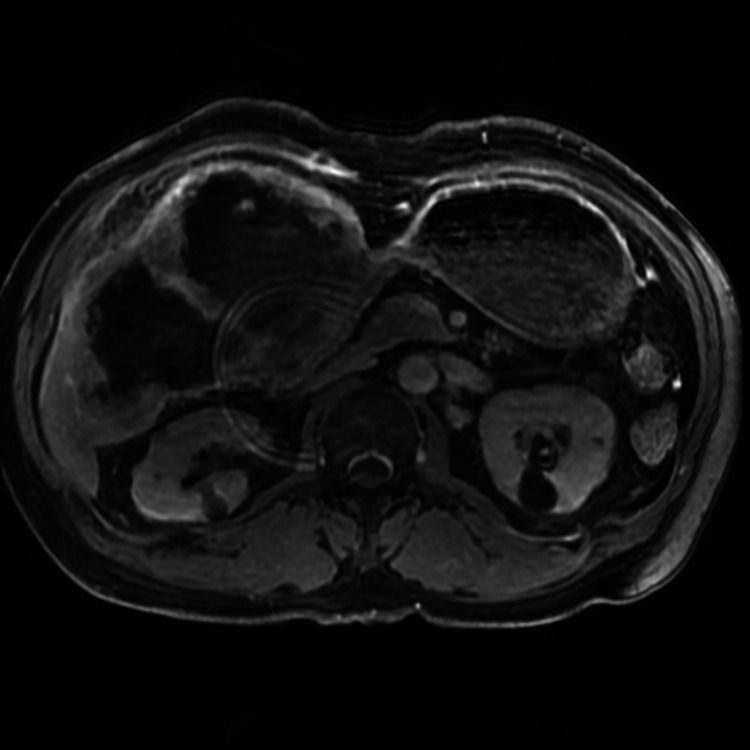
MRI Liver (axial view) showing large, heterogenous and extensively necrotic mass invading the liver with peripheral enhancement arising from the gallbladder. MRI: magnetic resonance imaging.

**Figure 4:**
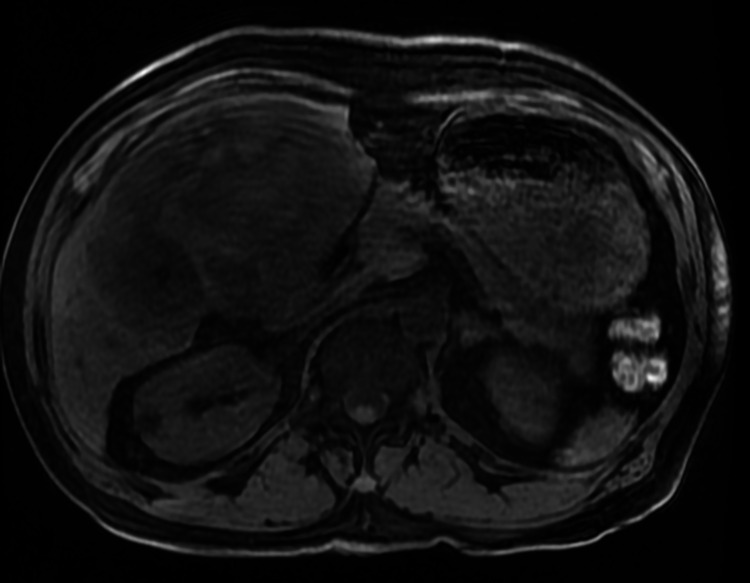
MRI liver (axial view) showing large heterogenous mass involving most of right hepatic lobe with extension into left lobe. MRI: magnetic resonance imaging.

Given systemic signs, the patient was started on broad-spectrum antibiotics, and cultures were sent. Interventional radiology was consulted for pigtail drains, and around 300 mL output was obtained, which was sterile with no bacterial growth, serosanguineous, and exudative by nature. Biopsy was also sent from the invasive mass of the gall bladder. Antibiotics were discontinued after successive negative blood cultures, but low-grade fever persisted. MRI liver was also obtained after interval placement of pigtails to delineate the mass further, which confirmed the aforementioned findings demonstrating large centrally necrotic heterogeneous mass and nodularity along with the omentum anteriorly with soft tissue signals representing peritoneal carcinomatosis, making it metastatic carcinoma of the gall bladder. Few porta hepatis nodes were measuring up to a centimeter on the short axis (Figures 3, 4).

**Figure 5:**
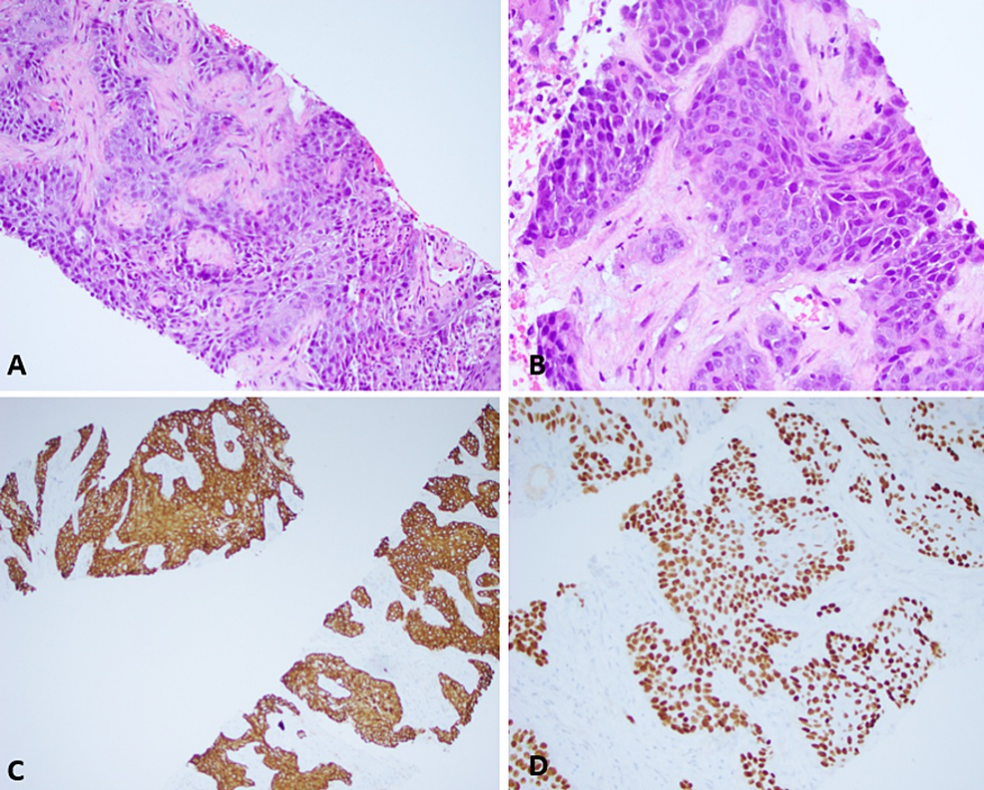
Carcinoma with squamous differentiation. This picture shows nests of neoplastic cells in specimen biopsy (H&E, A. 20× and B. 40×). Tumor cells are positive for CK5/6 (C) and P63 (D). H&E: hematoxylin and eosin.

Pathology results resulted in neoplastic cell proliferation with intercellular bridges and focal individual cell keratinization, with rare keratin pearls. Core biopsy did not show any glandular or hepatic parenchyma component. Immunohistochemical stains showed diffusely positive staining on neoplastic cells for p63, CK5/6, CK17, and CK19, while negative for hepatocyte, glypican-3, and arginase-1, supporting the diagnosis of squamous cell carcinoma (Figure 5A-D).

Given the current diagnosis, oncology, general surgery, and radiation oncology were taken on board. General surgery deemed the mass inoperable in this particular setting. Oncology recommended a trial of dose modified gemcitabine and cisplatin regimen versus dose reduced gemcitabine regimen for the patient. Given the severe risk of myelosuppression and the patient's attestation as Jehovah's witness and thus refusal of blood transfusion and considerable anemia below seven g/dl during the hospital course, oncological treatment was deferred. Radiation oncology scheduled the patient for palliative radiation for local control and tumor burden alleviation, but the patient opted for a hospice course.

## Discussion

Gallbladder cancer (GBC) is the most common biliary tract malignancy with the worse overall prognosis. Adenocarcinoma is the most frequent histological subtype accounting for over 80%-95% of cancer, adenosquamous variant accounting for 7%-10.6%. Pure squamous cell variety is exceedingly rare, accounting for about 2% to 3% of all GBC cases [1]. Compared to the other two variants, the pure squamous variant is more aggressive with complex biological behavior, including greater proliferation and local invasion capacity [1,2]. Because of their aggressive nature, invasion to neighboring areas, including liver, stomach, duodenum, or transverse colon, has been reported [1]. Squamous GBC usually spreads through lymphatic or hematogenous routes, although lymph node metastasis is rarely documented [3].

Even though reports of squamous GBC are rare, studies have tried to correlate clinicopathological differences amongst adenosquamous carcinoma and squamous variant. As per one retrospective study, pure SCC cases appeared to be well differentiated with substantial keratinization, including dyskeratotic cells and pearl formation. On the other hand, the adenosquamous variant appeared to be poorly differentiated with a high histological grade with an invasive pattern [1,4]. Despite having better differentiation and a lower rate of lymphatic invasion, squamous GBC is associated with a poorer prognosis than other variants [4]. Building interest and research on squamous GBC has recently shown CD109 as a novel marker for squamous GBC, a co-receptor for TGF-β1 (transforming growth factor-beta one) that increases TGF-β receptor endocytosis and, hence, negatively regulated TGF-β (transforming growth factor-beta) signaling. Another such marker of interest is FGFBP1 (fibroblast growth factor binding protein one) which is known to extend the biological activity of FGF (fibroblast growth factor) [5,6].

From the treatment perspective, surgical resection is the best bet for squamous GBC. Kalayarasan et al., in their retrospective study, showed that despite higher T (tumor size) staging, an R0 (complete) resection could be accomplished in most cases. After such resections, survival could be comparable to adenocarcinoma [7].

A handful of trials have been conducted for unresectable advanced biliary tract cancers (ABC), with the ABC-02 trial being the landmark for treatment (Table 1) [8-11].

**Table 1:** Trials notable for chemotherapy-naive and resistant advanced biliary tract carcinoma.

Trial	Phase	Regimen	Outcome
Notable trials for chemotherapy-naïve advanced biliary tract carcinoma			
ABC-02 (2010) NCT00262769 [8]	III	Gemcitabine + Cisplatin (GC) vs Gemcitabine (G) alone	Median overall survival: (in months) p<0.001 GC: 11.7 G: 8.1 HR: 0.64
			Progression-free survival: (in months) p<0.001 GC: 8.0 G: 5.0
Cisplatin/Gemcitabine with nab-Paclitaxel (2019) NCT02392637 [9]	II	GC + nab-Paclitaxel vs GC (historical data from ABC-02): non-randomized	Median overall survival: (in months) GC + nab-Paclitaxel: 19.2 (vs ABC-02)
			Progression free survival: (in months) GC + nab-Paclitaxel: 11.8 (vs ABC-02)
AMEBICA PRODIGE 38 (2019) NCT02591030 [10]	II	Modified FOLFIRINOX (mF) vs GC alone	Median overall survival: (in months) mF: 11.7 GC: 14.3
			Progression-free survival: (in months) mF: 6.2 GC: 7.4
Notable trial for GC-resistant advanced biliary tract carcinoma			
ABC-06 (2019) NCT01926236 [11]	III	FOLFOX + ASC (F-ASC) vs ASC alone	Median overall survival: (in months) 6.2 vs 5.3 (for F-ASC vs ASC) Overall Survival Rate: (%) At 6 months: 50.6 vs 35.5 At 12 months: 25.9 vs 11.4

In treatment naïve patients, it is recommended to utilize gemcitabine and cisplatin (GC) regimen [8]. The trial has been studied in one of the retrospective studies in the United States. It extended its spectrum to include patients with a performance status of above two and showed similar results as ABC-02. However, baseline CEA levels above three, stage IVB at diagnosis, and performance status above two were associated with poor outcomes [12]. Lately, clinical trial in phase II with GC and nab-Paclitaxel (owing to its improved stroma delivery) has shown some benefit over GC, but ABC-02 remains the gold standard for now until we have more evidence [8,9]. For those who have not demonstrated response to ABC-02, trial ABC-06 in phase III having FOLFOX with active symptom control (ASC) showed improved overall survival making it an effective second-line therapy [11].

## Conclusions

Squamous cell carcinoma in the biliary tract and specifically in the gallbladder is one of the rarest entities known in the oncological field of medicine. Given the scarcity, literature on its treatment is limited and is mainly met with hospice care given its extent of burden at presentation and aggressive course. Our patient presented with fever with pulmonary embolism and hypercalcemia, which resolved with appropriate management but accidentally found to have a massive liver abscess-like lesion. Given the high likelihood of malignancy, he underwent a biopsy which returned as squamous cell carcinoma of the gallbladder. Thus, the possibility of squamous cell carcinoma should be kept in mind while dealing with unusual radiological findings that do not match clinical presentations.
